# Spectrum of Cerebrovascular Disease in Patients with Multiple Myeloma Undergoing Chemotherapy—Results of a Case Control Study

**DOI:** 10.1371/journal.pone.0166627

**Published:** 2016-11-30

**Authors:** Archana Hinduja, Kaustubh Limaye, Rahul Ravilla, Appalnaidu Sasapu, Xenofon Papanikolaou, Lai Wei, Michel Torbey, Sarah Waheed

**Affiliations:** 1 Department of Neurology, University of Arkansas for Medical Sciences, Little Rock, AR, United States of America; 2 Department of Hematology and Oncology, University of Arkansas for Medical Sciences, Little Rock, AR, United States of America; 3 Multiple Myeloma for Research and Therapy, University of Arkansas for Medical Sciences, Little Rock, AR, United States of America; 4 Department of Neurology, Ohio State University Wexner Medical Center, Columbus, OH, United States of America; 5 Department of Neurosurgery, Ohio State University Wexner Medical Center, Columbus, OH, United States of America; Universitair Medisch Centrum Utrecht, NETHERLANDS

## Abstract

**Objectives:**

Patients with multiple myeloma (MM) are at increased risk of arterial thrombosis. Our aim was to determine the risk factors, mechanisms and outcome of strokes in these patients.

**Methods:**

We conducted a retrospective matched case–control study from our database of MM patients enrolled in Total Therapy (TT) 2, TT3a and TT3b protocols who developed a vascular event (transient ischemic attack, ischemic stroke, or intracerebral hemorrhage) from October 1998 to January 2014. Cases were matched for age-matched selected controls. Baseline demographics, risk factors, MM characteristics, laboratory values, and mortality of cases were compared to those of controls. Multivariate logistic regression analysis identified risk factors associated with stroke. Ischemic strokes (IS) were classified with modified Trial of Org 10172 in Acute Stroke Treatment (TOAST) criteria.

**Results:**

Of 1,148 patients, 46 developed a vascular event (ischemic stroke, 33; transient ischemic attack, 11; hypertensive intracerebral hemorrhage, 2). Multivariate logistic regression analysis determined renal insufficiency (odds Ratio, 3.528; 95% CI, 1.36–9.14; P = 0.0094) and MM Stages I and II (odds Ratio, 2.770, 95% CI, 1.31–5.81; p = 0.0073) were independent predictors of stroke. In our study, strokes attributable to hypercoagulability, atrial fibrillation and small-vessel occlusion were common mechanisms. After a stroke, 78% of patients were discharged to home or a rehabilitation facility and 4% to a long-term nursing facility; in-hospital mortality was 15%. Despite suffering a stroke no significant differences in survival were observed.

**Conclusion:**

In our cohort of multiple myeloma patients, renal failure and MM Stages I and II had increased risk of stroke.

## Background

Multiple myeloma (MM) is the second most common hematological malignancy among adults in the United States [1], and patients with MM have an increased risk of venous thromboembolism (VTE) [2]. Although the use of combination chemotherapy has markedly improved clinical outcomes for patients with multiple myeloma [3], these agents have been associated with an increased risk of venous thromboembolism, especially during the first months of chemotherapy. Factors that contribute to thrombosis include those associated with the patient, cancer and treatment [4–6]. Interestingly, increased rates of arterial thrombosis (coronary artery disease, cerebrovascular disease, myocardial infarction) have been reported, and the highest incidence occurs during induction of chemotherapy [7].

In a large population-based study (18,627 MM patients, 70,991 matched controls) the hazard ratios of VTE at 1, 5, and 10 years were 7.5, 4.6, and 4.1, and the risk of cerebrovascular disease was 1.5, 1.2, and 1.2 respectively [8]. Currently, there is limited data on the risk factors, mechanisms and outcomes of strokes among patients with MM. Knowledge of such predictors will help us adopt preventive strategies to reduce the incidence of stroke and improve outcomes in the near future. The primary aim of our study was to identify the stroke types, mechanisms, risk factors, and outcomes at discharge after an acute stroke among MM patients undergoing three similar chemotherapy regimens. In addition, we assessed the rates of recurrent strokes and survival time after the index event.

## Materials and Methods

### Study Population and Data Collection

Our study was retrospective review of our database of MM patients enrolled in Total Therapy (TT) 2, TT3a, and TT3b protocols and managed at our academic tertiary care medical center from October 1998 to January 2014; the institutional review board of University of Arkansas for Medical Sciences approved the study. Details of the protocols and patient outcomes were previously reported [9–12]. All patients signed an informed consent according to University of Arkansas for Medical Sciences and federal guidelines. All protocols were approved by our institutional review board of University of Arkansas for Medical Sciences and monitored by a data safety and monitoring board of University of Arkansas for Medical Sciences and outcome data were audited by an independent team of reviewers. Magnetic resonance imaging studies were performed in all patients who had symptoms and signs suggestive of a stroke. All patients who sustained an ischemic stroke (IS), transient ischemic attack (TIA) or intracerebral hemorrhage (ICH) from the time of enrollment until their last follow-up course were included.

Briefly, TT2 consisted of two arms of newly diagnosed MM patients who were ≤ 75 years old. At enrollment, patients were randomly assigned either to a control group (no thalidomide) or experimental group (thalidomide). Both arms consisted of multi-agent induction chemotherapy along with tandem autologous transplants, with the only difference in the inclusion or exclusion of thalidomide. Thalidomide doses were 400 mg daily during induction chemotherapy, 100 mg daily between transplantations, 200 mg daily with consolidation therapy, 100 mg daily during the first year of maintenance therapy, and then 50 mg on alternating days thereafter; the drug was given until relapse or adverse events occurred. Low-molecular-weight heparin was given prophylactically to all patients in the thalidomide group starting in July 2001.

Total Therapy 3 protocols were characterized by incorporation of bortezomib in addition multi-agent chemotherapy that consisted of thalidomide at induction and maintenance phases. TT3a and TT3b differed only in the maintenance phase; TT3a applied bortezomib, thalidomide and dexamethasone only in the first year and only thalidomide and dexamethasone thereafter, but TT3b applied bortezomib, lenalidomide and dexamethasone for all 3 years. Thus only the maintenance phase of TT3a and TT3b deferred with TT3a having thalidomide and TT3b with lenalidomide. Thalidomide dosage did not exceed 200 mg in any phase of the protocols, and titration of dosage to a minimum of 50 mg was permitted to ensure maximum compliance to the treatment. For lenalidomide, the maximum and minimum permitted dosages at the time were 15 mg and 5 mg, respectively. No routine prophylaxis for deep vein thrombosis was administered in the TT3 protocols. Of note, renal insufficiency was not an exclusion criterion for TT2 as long as it was of recent onset and due to Bence-Jones proteinuria or hypercalcemia, but patients were not eligible for TT3 protocols if creatinine levels were >3 mg/dl, regardless of the reason. We specifically selected patients enrolled in TT2 and TT3 protocols because wanted to study the effects of thalidomide and lenalinomide exclusively. This cut of makes it feasible to include patients with mild to moderate renal failure. Thus varying degrees of renal insufficiency was prevalent in all treatment protocols.

All cases (i.e., MM patients who had strokes) were compared with age-matched controls (i.e., MM patients who did not have strokes). Baseline demographics (age, gender, race), risk factors (hypertension, hyperlipidemia, diabetes mellitus, coronary artery disease, congestive heart failure, atrial fibrillation, obstructive sleep apnea, smoking, alcohol use, other malignancies, renal insufficiency, need for hemodialysis, prior VTE), MM characteristics (protocol, stage, isotype, risk category), laboratory values (complete blood count, coagulation profile, fasting blood glucose, β2 microglobulin, blood urea nitrogen, creatinine, albumin), and mortality were compared between both groups. Additionally, among the cohort of stroke cases, baseline laboratory values at the initial visit were compared with those during the vascular insult.

### Clinical Definitions

Transient ischemic attack was defined as rapidly developing symptoms and signs of loss of cerebral function of vascular origin with a negative diffusion-weighted imaging (DWI) sequence on magnetic resonance imaging (MRI) [13] and ischemic stroke was defined as a positive DWI sequence of MRI. The stroke subtype was classified based on the TOAST (Trial of Org 10172 in Acute Stroke Treatment) criteria by two neurologists independently, and in case of disagreement settled by a third neurologist [14]. Conclusions of the stroke subtype were drawn based on the laboratory data, imaging studies, cardiac studies (transthoracic and transesophageal echocardiographic, telemetry monitoring data in the medical records). Intracerebral hemorrhage (ICH) was defined as extravasation of blood within the brain parenchyma; intracerebral hemorrhage secondary to arteriovenous malformation, trauma, tumor, or cerebral venous sinus thrombosis was excluded. Hypertension was defined as systolic blood pressure >140 mm Hg, diastolic blood pressure >90 mm Hg, or taking an antihypertensive agent. Diabetes mellitus was defined as fasting blood glucose ≥ 125 mg/dl or taking an oral hypoglycemic medication. Hyperlipidemia was defined as low-density lipoprotein >160 mg/dl, total cholesterol > 240 mg/dl, or taking lipid-lowering medication. Smoking was defined as current smokers or smokers who quit within the previous 12 months. Alcohol abuse was defined as daily drinking >2 drinks for >1 year. Renal insufficiency was defined as an increase in serum creatinine, decline in glomerular filtration rate, or decreased urine output; dose adjustments to chemotherapeutic agents were made based on creatinine levels.

### Clinical Outcomes

The disposition at discharge and recurrence of vascular event upon follow up were assessed in the cohort of stroke cases (i.e., MM patients who had strokes). In addition, all-cause mortality and survival upon follow up was compared between both groups.

### Statistical Analysis

Baseline characteristics, risk factors, laboratory parameters, and outcomes of the two groups were compared with Pearson’s Chi-square test or Fisher’s exact test for categorical variables and student *t-*test for continuous variables. All parameters determined by univariate analysis to be significant (*P*<0.05) were included in a multivariate regression analysis to identify the risk factors associated with stroke in MM patients. Among the cohort of stroke cases, comparison of the laboratory values between initial visit and during the vascular insult was performed using the paired *t*-tests. Overall survival was calculated from the start of the protocol to death from any cause. Patients who were still alive in April 2015 were censored at the date of last contact. Survival curves (estimated using the method of Kaplan-Meier) of cases and controls were compared with the log-rank test; estimated median with 95% confidence intervals were reported. Survival curves were used to estimate mortality rates for both groups and were estimated at 2, 3, 4, and 5 years. For all statistical analyses, SAS 9.4 software was used, and, *P ≤*0.05 was considered statistically significant.

## Results

**Fig 1:**
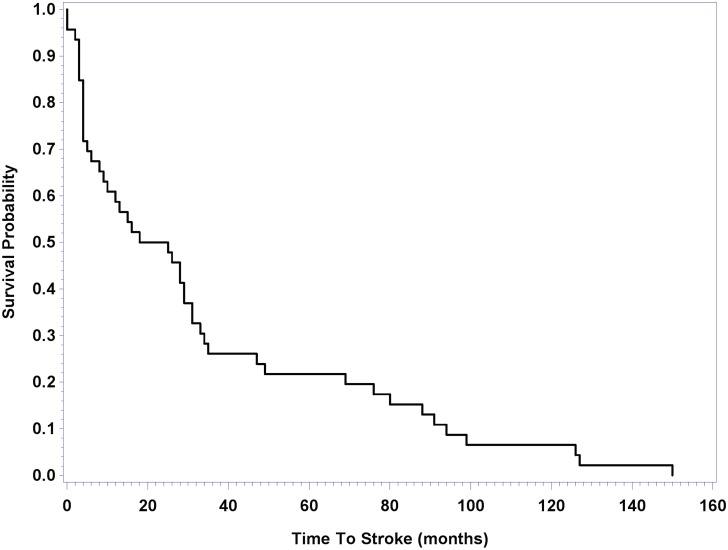
Kaplan-Meier curve representing the time from enrollment to stroke only in the cohort of MM stroke cases.

During this study period, 1,148 patients with MM were enrolled; TT2 without thalidomide (TT2−Thal; *n* = 345), TT2 with thalidomide (TT2+Thal; *n* = 323), TT3a (*n* = 303), and TT3b (n = 177); 4% (46 patients) of patients developed an acute stroke after enrollment with a median time of 21.5 months (0–150 months, Fig 1).

IS was the most common type of stroke (72%; 33 patients) followed by transient ischemic attack (24%; 11 patients), and hypertensive basal ganglia hemorrhage (4%; 2 patients). All patients diagnosed as TIA in our cohort had a negative DWI on MRI. The most common mechanisms of stroke in our cohort of MM patients was thrombotic in nature from hypercoaguable state (15%; 7 patients), followed by cardioembolism (13%; 6 patients), small vessel disease (13%; 6 patients), multiple other known mechanisms (watershed patterns of strokes from hypotension, sepsis) (15%; 7 patients), large-vessel disease (7%; 3 patients), and cryptogenic in (9%; 4 patients). Inter current systemic infections were observed in 28% (13 patients) of stroke patients but none of them had evidence of nonbacterial thrombotic endocarditis. Vascular events of ischemic etiology occurred despite prior use of antiplatelet and anticoagulation therapy (two patients in each group). After a vascular event, antiplatelet agents were administered to 16 patients and anticoagulants to 7 patients; however 23 patients were ineligible for both antiplatelet and anticoagulant agents due to thrombocytopenia.

Demographics, risk factors, and baseline laboratory values are described in Tables 1 and 2. Univariate analysis indicated that patients who developed strokes were more likely than controls to have a positive smoking history (26.1% vs 13%, *P* = 0.0381), have renal insufficiency (23.9% vs 8.0%, *P* = 0.0039), require hemodialysis (10.9 vs 0.7%, *P* = 0.004) and have Stage I or II myeloma as opposed to Stage III (Stage I: 23.9% vs 9.4%; Stage II: 17.4% vs 12.3; Stage III: 58.7% vs 78.3%; *P* = 0.025). These factors remained significantly positive when hemorrhagic strokes were excluded from analysis. Since only one patient had a prior stroke, this risk factor was excluded from analysis. Multivariate logistic regression analysis (Table 3) indicated that independent predictors associated with stroke were renal insufficiency (odds ratio, 3.528; 95% CI, 1.36–9.14; *P* = 0.0094) and Stage I and II myeloma (odds ratio, 2.770; 95% CI, 1.31–5.81; *P* = 0.0073); significance of smoking was borderline (odds ratio, 2.324; 95% CI, 0.98–5.54; *P* = 0.0572). Hemodialysis was excluded from this analysis because only one patient in the control group required hemodialysis.

**Table 1:** Characteristics of Multiple Myeloma Patients With and Without Stroke.

	Stroke (N = 46)*	No Stroke (N = 138)†	*P* value
Age, years	60.6 (7.7)	60.7 (7.8)	0.8960
Women	23 (50.0)	57 (41.6)	0.3023
Race, Caucasian	44 (95.7)	125 (90.6)	0.3635‡
Hypertension	25 (54.4)	60 (43.5)	0.2003
Hyperlipidemia	15 (33.3)	36 (26.1)	0.3464
Diabetes mellitus	8 (17.4)	13 (9.4)	0.1409
Coronary artery disease	5 (10.9)	15 (10.9)	>0.99
Congestive heart failure	2 (4.4)	12 (8.7)	0.5237‡
Atrial fibrillation	8 (17.4)	13 (9.4)	0.1409
Transient ischemic attack	2 (4.4)	0	0.0615‡
Obstructive sleep apnea	0	2.2 (3)	0.5743‡
Smoking	12 (26.1)	18 (13.0)	0.0381
Alcohol abuse	1 (2.2)	2 (1.5)	>0.99‡
Other malignancy	4 (8.7)	22 (16.1)	0.2159
Renal insufficiency	11 (23.9)	11 (8.0)	0.0039
Hemodialysis	5 (10.9)	1 (0.7)	0.0040‡
Prior deep vein thrombosis	13 (28.9)	27 (19.6)	0.1888
Protocol			
T2 TT2 with Thalidomide TT2 without Thalidomide TT3a TT3b	25 (54.4) 17 (37.4) 8 (17.4) 15 (32.6) 6 (13.0)	78 (55.6) 55 (39.9) 23 (16.7) 30 (21.7) 30 (21.7)	0.3787
MM stage			
I II III	11 (23.9) 8 (17.4) 27 (58.7)	13 (9.4) 17 (12.3) 108 (78.3)	0.0182‡
MM isotype			
IgG IgA FLC-κ FLC-λ Other	27 (58.7) 10 (21.7) 4 (8.7) 5 (10.9) 0	68 (49.3) 34 (24.6) 14 (10.1) 15 (10.9) 7 (5.1)	0.6128‡
MM risk	–0.13 (0.61)	0.09 (0.67)	0.0941

Values are expressed as n (%), mean ± standard deviation as appropriate

*Hyperlipidemia and for Prior deep vein thrombosis, N = 45; for MM risk, N = 37

^†^Other malignancy, N = 137; for MM risk, N = 88

^‡^Fisher’s exact test

**Table 2:** Baseline Laboratory Values of MM Patients With and Without Stroke.

	Stroke (N = 46)*	No Stroke (N = 138)†	*P* value
Hemoglobin, g/dl	11.4 (1.8)	11.4 (1.9)	0.9135
Hematocrit, %	34.3 (5.3)	34.1 (5.7)	0.8519
Platelets, 10^9^/L	258.7 (99.2)	247.7 (101.7)	0.5230
Prothrombin time, sec	13.4 (1.5)	13.4 (1.8)	0.9240
APTT, sec	30.5 (6.5)	28.7 (4.5)	0.0881
International normalized ratio	1.07 (0.12)	1.09 (0.15)	0.5321
Serum glucose, mg/dl	105.5 (25.8)	107.2 (30.9)	0.7359
Beta 2-microglobulin, mg/L	5.2 (7.0)	4.6 (3.8)	0.5783
Fibrinogen, mg/dl	428 (159)	406 (136)	0.4534
Creatinine, mg/dl	1.3 (1.0)	1.2 (1.0)	0.5855
Lactate dehydrogenase, U/L	166.8 (47.8)	170.0 (75.9)	0.7627
Serum albumin g/dl	3.93 (0.52)	3.95 (0.61)	0.7797
White blood cell count, 10^3^/dl	5.9 (2.4)	6.1 (3.1)	0.7138

Values represented as n (%), mean ± standard deviation;

APTT, activated partial thromboplastin time

*Fibrinogen, N = 36

^†^Beta 2-microglobulin, N = 137; Fibrinogen, N = 63

**Table 3:** Multivariate Analysis of Risk Factors Associated with Stroke.

	Unadjusted		Adjusted		*P* value
	Odds Ratio	95% CI	Odds Ratio	95% CI	
Smoking	2.35	1.03–5.36	2.324	0.98–5.54	0.0572
Nephropathy	3.63	1.45–9.07	3.528	1.36–9.14	0.0094
MM Stage I and II	2.53	1.24–5.17	2.77	1.31–5.81	0.0073

**Fig 2:**
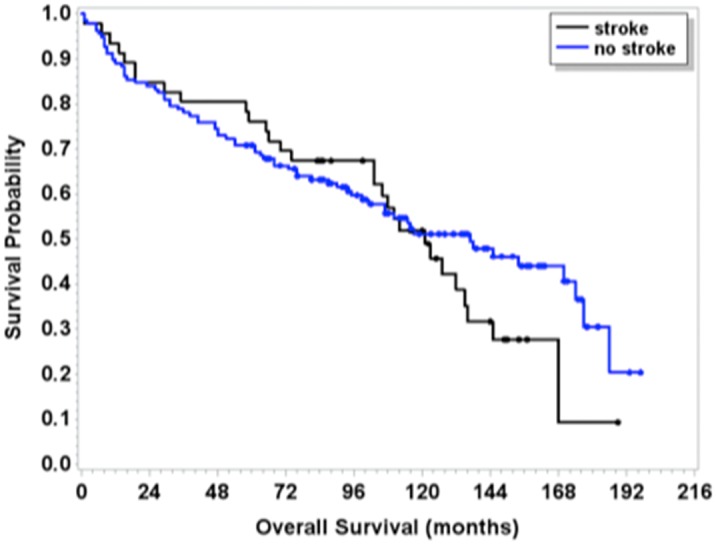
Overall survival (OS) was calculated from the start of the protocol to death from any cause. Patients who were still alive were censored at the date of last contact. Survival curves were estimated using the method of Kaplan-Meier.

In our cohort of 46 cases, 78% (36 patients) were discharged home or to a rehabilitation facility and 4% (2 patients) to a long-term nursing facility. In-hospital mortality was 15% (7 patients), with stroke-related mortality in 13% (6 patients). The two groups did not differ significantly in median overall survival (121 vs 137 months, *P* = 0.34) or mortality rate at 2 years (15.2% vs 16.1%, *P* = 0.89), 3 years (19.6% vs 21.9%, *P* = 0.73), 4 years (19.6% vs 27.0%, *P* = 0.29) and 5 years (23.9% vs 29.2%, *P* = 0.47) (Fig 2). During a median follow up of 10 years, the cumulative risk of recurrent stroke was 15% (6/39 patients). Among the stroke cases, there was no significant relationship between mortality and use of thalidomide; median survival was 103 months for a thalidomide-based regimen (95% CI 42,135) and 78 months (95% CI 10,115)(*P* = 0.1808) for a regimen without thalidomide.

Although baseline laboratory values (Table 4) did not significantly differ between the groups, the cohort of stroke cases had a significant decrease in platelet count (112.6±78.6 vs 255.2±104.2, *P*<0.0001) and increase in International Normalized Ratio (1.25±0.33 vs 1.08±0.12, *P* = 0.0096) during the vascular insult, as compared to baseline values.

**Table 4:** Laboratory Values at Baseline and During Vascular Event for MM Patients Who Experienced Stroke.

Variable	N	Baseline	Stroke	*P* value
Hemoglobin, g/dl	35	11.4 (1.9)	11.3(1.8)	0.7906
Hematocrit, %	35	34.5 (5.6)	34.2 (5.5)	0.8138
Platelets, 10^9^/L	35	255.2 (104.2)	112.6 (78.6)	<0.0001
Prothrombin time, sec	30	13.5 (1.5)	14.9 (2.8)	0.0145
APTT, sec	28	31.1 (5.9)	32.2 (8.4)	0.4959
International normalized ratio	30	1.08 (0.12)	1.25 (0.33)	0.0096
Serum glucose, mg/dl	30	108.4 (28.4)	113.9 (30.5)	0.4394

Values represented as n (%), mean ± standard deviations as appropriate

APTT, activated partial thromboplastin time

Observations with missing data were dropped in Table 4. This was done on a variable-by-variable basis, which is why the sample size changes depending on the variable being analyzed.

## Discussion

This large single-center retrospective, comparative case−control study of MM patients enrolled in TT2, TT3a, and TT3b protocols over 10 years, identified 4% of patients who developed an acute stroke. Only 4% of the strokes were of the hemorrhagic type, which is different from the general population (10%) and patients with cancer (14–18%) [15–17]. Although prior studies demonstrated increased risk of arterial thrombosis from activated prothrombotic factors, especially during the induction phase of anti-MM therapy [7, 18], vascular events occurred even months after the induction phase in our cohort. The major mechanisms of strokes in our cohort were from underlying hypercoagulable state or chemotherapeutic agents, cardioembolic causes and small-vessel occlusion. These are a bit different compared to the general population where strokes from large vessel disease, small vessel disease and cardioembolic are predominant mechanisms, and amongst patients with cancer, besides these, cryptogenic and embolic phenomenons are common mechanisms [19–23]. Although in-hospital mortality was higher in our cohort compared to the national surveillance standards, which is 5% [24], underlying malignancy is a well-known predictor of mortality [9, 25]. Despite these odds, the percentage of patients discharged home, to acute rehabilitation facilities and to long-term nursing facilities after a stroke was similar to the national average [24]. The annual risk of recurrent strokes in the general population is about 5% [15, 26]. Underlying malignancy is a predictor of recurrent strokes with a yearly recurrence rate of 15% [21, 27]. Although the recurrence rate of stroke amongst patients with multiple myeloma is not known, the cumulative risk of recurrence in our cohort over a decade of follow up was only 15%.

The major risk factors associated with stroke in our study were renal insufficiency and Stage I or II MM, with a trend for smoking, which is a novel finding when compared to prior results, indicating that hypertension and smoking were significant risk factors [7]. Approximately 20−40% of newly diagnosed MM patients have renal impairment, explaining the large contribution of this mechanism in our cohort [28, 29]. Although no significant differences in their baseline renal functions were observed, renal insufficiency was a significant predictor of stroke thus reflective that these developed during the disease course. Large population- based studies have shown increased risk of stroke, asymptomatic cerebral lacunae, and recurrent strokes from renal dysfunction, thereby explaining the heightened risk of stroke among MM patients [30–32]. Patients with kidney disease are at increased risk of cardiogenic and atherosclerotic strokes, which may partly explain the noncancer-related mechanism in our cohort [33]. Increased mortality and worse outcomes have been observed in stroke patients with concurrent kidney disease, and this may partly have contributed to the high in-hospital morality observed in our study [34, 35]. Although kidney disease and stroke share common risk factors, including hypertension, hyperlipidemia, diabetes mellitus, smoking, obesity, and aging, these factors were not significant predictors in our cohort. Patients with kidney disease have (a) non traditional risk factors (increased inflammation from oxidative stress, asymmetric dimethylarginine that inhibits nitric oxide, hyperhomocysteinaemia, sympathetic over activity triggering vascular injury and endothelial dysfunction) and (b) uremia risk factors (uremic toxins, sodium and water retention, anemia and malnutrition, abnormal calcium and phosphorous metabolism, hyperparathyroidism and decreased Klotho protein expression) that contribute to increased cardiovascular risk [36–41]. Additionally the kidney and the brain share similar susceptibilities to hemodynamic derangements [42]. Also noteworthy, patients with MM who developed renal insufficiency had worse clinical outcomes despite improvement in their renal function or lack of significant difference in their baseline renal functions between various treatment protocols [43]. Thus increased risk of stroke, recurrent stroke, and mortality in our cohort could partly be due to renal disease, which may or might not have resulted from myeloma. Although systemic infections were identified in a third during the vascular event and systemic infection is a well-identified triggering mechanism of stroke [44], we did not analyze the association with stroke because of the retrospective nature of our study.

Increased risk of stroke was observed in patients with Stage I and II MM, but no such relationship was observed for various treatment protocols or thalidomide use. However, the relationship with thalidomide may be underpowered given the case-control nature of our study. No study has yet evaluated the risk of VTE and arterial thrombosis for patients with Stage 1 or II MM; differences have only been noted only for patients with newly diagnosed MM versus those with relapsed MM [45, 46]. Thalidomide alone does not increase the risk of VTE, and incidence of VTE was 3−5% for patients with newly diagnosed MM [3, 47]. The combination of thalidomide with dexamethasone significantly increased the incidence of VTE to 14–28% in newly diagnosed MM patients [5, 45, 48] and thalidomide in combination with multiagent chemotherapy increases VTE risk to 16−34% in newly diagnosed patients[9, 49]. Similarly, lenalinomide did not increase the VTE risk as a single agent, but in combination with dexamethasone, risk increased to 8−75% in newly diagnosed MM patients [3, 50], and the increased risk of VTE was highest in the first year after diagnosis. MM patients have an increased risk also of arterial thromboembolic events and thrombosis is associated with inferior survival for these patients [51]. Although in-hospital mortality was high, it was not statistically significant, and overall survival was not significantly different in our analysis of matched cases and controls. These results are discordant with those of large population-based studies probably because we had a smaller sample size [51]. In our study, patients developed strokes at different time frames from enrollment into various protocols, which also accounted for mortality at various time frames. Recent literature has clearly demonstrated that low body weight, thrombocytopenia and renal failure are strong risk factors associated with early mortality from infections and various comorbidities [52]. Other variables that have been associated with mortality among myeloma patients include age, worsening stage and type of therapy [53, 54]. The mechanism of early mortality in both groups was from their inherit risk through various processes, with stroke being the culprit in the cases and other causes in the controls, thereby explaining the similarities in early survival in both groups.

Management of strokes in patients with MM depends upon the etiology. Common mechanisms of stroke in patients with underlying malignancy include hypercoagulability and cardioembolism, among other mechanisms [17, 18, 23]. Interestingly, our patients developed strokes despite a trend towards coagulopathy, to the extent that 50% were ineligible for immediate use of antiplatelet agents. In our study, besides strokes attributed to coagulopathy, other mechanisms such as atrial fibrillation and small-vessel occlusion played major contributing mechanisms. These results have heightened our awareness that the incidence of stroke in patients with renal insufficiency may be decreased with some aggressive preventive measures, including management of hypertension, diabetes mellitus, hyperlipidemia; avoidance of hypotension; and use of novel chemotherapeutic agents.

Some major drawbacks of our study include a retrospective review, referral bias rather than true population patterns, inclusion of a subset of MM patients enrolled in specific treatment protocols, aggressiveness of the myeloma during the index event, a small sample size, and a case−control analysis. Because of a small sample size, the results of our study may need to be interpreted with caution until these are validated by large studies. Although the stroke subtype was arrived based on the TOAST criteria, the lack of long-term cardiac monitoring evaluation may have possibly underestimated the percentage of cardioembolic strokes. Besides, certain patients had multiple factors in which case the imaging pattern and triggering factor were the deciding factors. Although nephropathy preceded the occurrence of stroke, the exact time frame and the degree of renal failure was unavailable because definitions varied over the entire decade. Finally, the etiology of renal disease may either be due to myeloma or non-myeloma related origin and could not be evaluated because of the retrospective nature of our analysis.

To conclude, in our cohort of multiple myeloma patients, renal failure, MM Stage I and II were significant risk factors associated with stroke, which is different when compared to the general population. Despite a high rate of in-hospital mortality, patients with MM who develop a vascular event have good outcomes at discharge and overall long-term survival. Besides myeloma- related thrombosis, cardioembolic causes and small-vessel occlusions were other possible stroke mechanisms. Unlike other malignancies, patients with MM had greater risk of stroke if they had renal disease; thus renal disease may serve as a marker to identify high-risk patients, but will need confirmation in large population-based studies. Adoption of various preventive measures, especially for patients with renal insufficiency, may reduce the risk of stroke in future.

## Supporting Information

S1 Table Characteristics of Multiple myeloma patients with Ischemic stroke and controls. (DOCX)

S2 Table Comparison of baseline laboratory values between MM patients experiencing a ischemic stroke with control. (DOCX)
