# Correction: Spectrum of Cerebrovascular Disease in Patients with Multiple Myeloma Undergoing Chemotherapy—Results of a Case Control Study

**DOI:** 10.1371/journal.pone.0175218

**Published:** 2017-03-30

**Authors:** Archana Hinduja, Kaustubh Limaye, Rahul Ravilla, Appalanaidu Sasapu, Xenofon Papanikolaou, Lai Wei, Michel Torbey, Sarah Waheed

The fourth author’s name is spelled incorrectly. The correct name is Appalanaidu Sasapu.
