# Supplementary material for: Spectrum of Cerebrovascular Disease in Patients with Multiple Myeloma Undergoing Chemotherapy—Results of a Case Control Study
Source: PLoS One. 2016 Nov 30;11(11):e0166627. doi: 10.1371/journal.pone.0166627 (PMC5130211; doi:10.1371/journal.pone.0166627)
Supplement: S1 Table — (DOCX) [file pone.0166627.s001.docx]

**S1 Table.** Characteristics of Multiple myeloma patients with Ischemic stroke and controls

| **Variable** | **Ischemic**  **Stroke**  **(N=44)** | **No Stroke**  **(N=138)** | **P Value** |
| --- | --- | --- | --- |
| Age, years | 60.6 (7.7) | 60.7 (7.8) | 0.9368 |
| Women | 22 (50.0) | 57 (41.6) | 0.3109 |
| Race, Caucasian | 42 (95.5) | 125 (90.6) | 0.5284* |
| Hypertension | 23 (52.3) | 60 (43.5) | 0.3078 |
| Hyperlipidemia | 14/43 (32.3) | 36 (26.1) | 0.4073 |
| Diabetes mellitus | 8 (18.2) | 13 (9.4) | 0.1132 |
| Coronary artery disease | 5 (11.4) | 15 (10.9) | 0.9273 |
| Congestive heart failure | 2 (4.6) | 12 (8.7) | 0.5232 |
| Atrial fibrillation | 8 (18.2) | 13 (9.4) | 0.1132 |
| Transient ischemic attack | 1 (2.3) | 0 | 0. 2418* |
| Obstructive sleep apnea | 0 | 3 (2.2) | >0.99* |
| Smoking | 12 (27.3) | 18 (13.4) | 0.0268 |
| Alcohol abuse | 1 (2.3) | 2 (1.5) | 0.5664* |
| Other malignancy | 3 (6.8) | 22/137 (16.1) | 0.1222 |
| Renal insufficiency | 10 (22.7) | 11(8.0) | 0.0076 |
| Hemodialysis | 5 (11.4) | 1 (0.7) | 0.0034* |
| Prior Deep vein thrombosis | 13/43 (30.2) | 27 (19.6) | 0.1410 |
| Protocol  TT2  TT3a  TT3b | 23 (52.3)  15 (34.1)  6 (13.6) | 78 (56.5)  29 (21.0)  30 (21.7) | 0.2731 |
| MM Stage  I  II  III | 10 (22.7)  8 (18.2)  26 (59.1) | 13 (9.4)  17 (12.3)  108 (78.3) | 0.0279* |
| MM Isotype  IgG   IgA  FLC-κ    FLC-λ  Other | 25 (56.8)  10 (22.7)  4 (9.1)  5 (11.4)  0 | 68 (49.3) 34 (24.6) 14 (10.1)  15 (10.9)  7 (5.1) | 0.6898* |
| MM Risk, mean (SD) | -0.09 (0.61; N=35) | 0.09 (0.67; N=88) | 0.1760 |

Values are represented as n (%), means and standard deviation

*Fisher’s exact test
