# Supplementary material for: Spectrum of Cerebrovascular Disease in Patients with Multiple Myeloma Undergoing Chemotherapy—Results of a Case Control Study
Source: PLoS One. 2016 Nov 30;11(11):e0166627. doi: 10.1371/journal.pone.0166627 (PMC5130211; doi:10.1371/journal.pone.0166627)
Supplement: S2 Table — (DOCX) [file pone.0166627.s002.docx]

**S2 Table**. Comparison of baseline laboratory values between MM patients experiencing an ischemic stroke with control

| **Variable** | **Stroke**  **(N=46)** | **No Stroke**  **(N=138)** | **P value** |
| --- | --- | --- | --- |
| Hemoglobin, g/dl | 11.3 (1.8) | 11.4 (1.9) | 0.8415 |
| Hematocrit, % | 34.3 (5.3) | 34.1 (5.7) | 0.9257 |
| Mean corpuscular volume, fL | 95.0 (5.8) | 94.2 (5.2) | 0.4047 |
| Platelets, 10^9^/L | 255.9 (100.4) | 247.7 (101.7) | 0.6407 |
| Prothrombin time, sec | 13.4 (1.5) | 13.4 (1.8) | 0.9259 |
| APTT, sec | 30.3 (6.5) | 28.7 (4.5) | 0.1446 |
| International Normalized Ratio | 1.07 (0.12) | 1.09 (0.15) | 0.5674 |
| Serum glucose, mg/dl | 106.0 (26.2) | 107.2 (30.9) | 0.8187 |
| Beta 2-microglobulin, mg/L | 5.4 (7.2) | 4.6 (3.8; N=137) | 0.5246 |
| Fibrinogen, mg/dL | 432 (162; N=34) | 405.5 (136; N=63) | 0.3834 |
| Creatinine, mg/dL | 1.3 (1.0) | 1.2 (1.0) | 0.5613 |
| Lactate Dehydrogenase, U/L | 168.8 (47.8) | 169.7 (75.7) | 0.9038 |
| Serum Albumin g/dL | 3.93 (0.51) | 3.95 (0.61) | 0.8267 |
| White Blood cell count, 10^3^/dL | 5.9 (2.4) | 6.1 (3.1) | 0.7742 |

APTT Activated partial thromboplastin time
